# Another 10 years of PLOS Computational Biology: A data-driven reflection on trends in genomics research

**DOI:** 10.1371/journal.pcbi.1014471

**Published:** 2026-07-02

**Authors:** Jean Fan

**Affiliations:** Department of Biomedical Engineering, Johns Hopkins University, Baltimore, Maryland, United States of America; Johns Hopkins University, UNITED STATES OF AMERICA

## Abstract

Since the founding of *PLOS Computational Biology* 20 years ago, genomics research has advanced at a remarkable pace. In this 20th anniversary commentary, as an Editor for the Journal Section of Genomics, Epigenomics, & Proteomics, I take a data-driven dive into genomics research at *PLoS Computational Biology* by analyzing all submitted research papers in this section since 2017. This time window reflects the limits of the Editorial Manager records we were able to assemble, but it also coincides approximately with my own independent journey in this field. While this time window does not capture the journal’s full 20-year history, I hope this analysis will offer a data-driven reflection on how genomics research within the journal has evolved and provide a putative trajectory on how the field will surely continue to grow into the future.

## Trends in computational biology paper submissions highlight increasing popularity in genomics research in recent years

**Fig 1:**
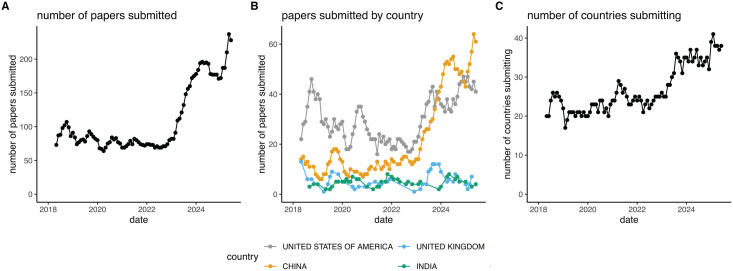
Trends in paper submissions to *PLOS Computational Biology* in Genomics, Epigenomics, & Proteomics. **(A)** Number of papers submitted over time per 6-month rolling window. **(B)** Number of papers submitted per country over time per 6-month rolling window. Country is based on the location of the corresponding author’s institutional affiliation. Top 6 countries by total submission shown. **(C)** Number of unique countries represented among submitted papers over time per 6-month rolling window.

From 2017 to 2025, approximately 1700 papers were submitted to *PLOS Computational Biology* under the Journal Section of Genomics, Epigenomics, & Proteomics. Aggregating these submissions into rolling 6-month windows, we can observe a pronounced surge in submissions in recent years, particularly since 2023 (Fig 1A). If we further break down number of submissions by the country, we note that while submissions have been historically dominated by the United States, China has recently overtaken as the country with the most paper submissions (Fig 1B). The trend likely reflects China’s significant funding investments in genomics research and exemplifies how government commitment can accelerate scientific output to shape the global research landscape [1]. In general, computational biology research in genomics has continued to grow into an increasingly global endeavor. If we count the number of different countries submitting papers per quarter, we again observe a pronounced increase in recent years (Fig 1B). This growing international submission pool highlights the need for a matched growth in community engagement such as through participation in peer review while maintaining consistent and rigorous standards. Likewise, such growth highlights opportunities for training and collaboration, data generation and sharing, as well as the development of tools and standards that can support a diverse and globally distributed scientific community.

## Keywords in submitted papers highlight how research trends in computational biology have been shaped by technological and algorithmic advances as well as public-health events

**Fig 2:**
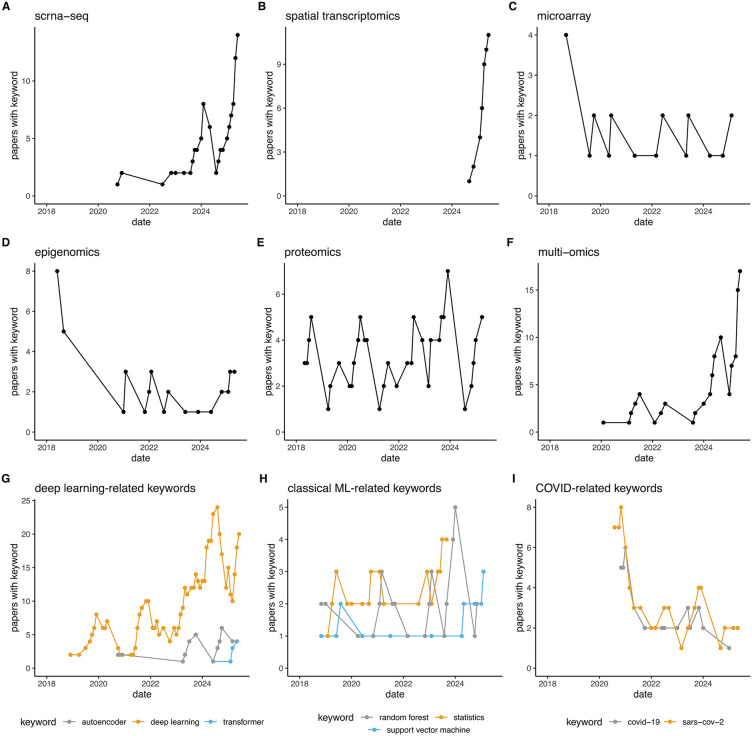
Trends in paper submissions keywords to *PLOS Computational Biology* in Genomics, Epigenomics, & Proteomics. Number of papers submitted over time with the keyword **(A)** “scrna-seq,” **(B)** “spatial transcriptomics,” **(C)** “microarray,” **(D)** “epigenomics,” **(E)** “proteomics,” **(F)** “multi-omics,” **(G)** various deep-learning method-associated keywords, **(H)** various classical machine-learning method-associated keywords, and **(I)** COVID-related keywords per 6-month rolling window.

To appreciate how research trends in computational biology have shifted over the years, we can analyze keyword usage over time among submitted papers (Fig 2). First, keywords related to advances in genomics technologies such as “scRNA-seq” and “spatial transcriptomics” have risen sharply in recent years, mirroring the widespread commercialization and adoption of these high-throughput transcriptomic profiling technologies and the subsequent demand for specialized computational tools to analyze the associated high-dimensional datasets (Fig 2A and 2B). In contrast, “microarray” as a keyword has remained stagnant despite overall increases in submissions, suggesting a decline in the field’s research emphasis on this technology (Fig 2C). Similarly, keywords such as “epigenomics” and “proteomics” have decreased in relative frequency, while “multi-omics” has shown a notable increase, reflecting a potential shift toward integrative approaches that combine diverse molecular modalities to achieve a more comprehensive understanding of biological systems (Fig 2D–2F). Beyond technological advancements, algorithmic advancements in deep learning have resulted in recent growth in its keyword usage, along with the emergence of specific model architectures such as “autoencoder” and “transformer” as keywords (Fig 2G). In contrast, classical machine learning approaches in “statistics” using model architectures such as “random forest” and “support vector machine” as keywords have remained relatively stagnant (Fig 2H), highlighting the broader impact of the advancements in deep learning artificial intelligence (AI) on all aspects of computational applications including in genomics research. Within this analyzed time window, the coronavirus disease 2019 (COVID-19) and associated severe acute respiratory syndrome coronavirus 2 (SARS-CoV-2) emerged to profoundly impact global public health. As researchers rapidly mobilized to study this novel disease, which was declared as a pandemic in early 2020, the frequency of “sars-cov-2” and “covid-19” as keywords in submitted manuscripts likewise peaked in early 2020 (Fig 2I). Although the frequency of “sars-cov-2” and “covid-19” as keywords in submitted manuscripts has since declined, it remains above pre-pandemic levels, highlighting ongoing efforts to better understand the disease to both improve treatments for those suffering from post-acute sequelae as well as in preparation of future pandemics. Although these trends are based on a limited number of total submitted papers, the general patterns underscore the close interplay between technological and algorithmic advances and public-health events with the evolving priorities of computational biology research in genomics.

## From publication to impact: Well-maintained open-source software lead in citations

If we look closer at individual papers, focusing on the most well-cited *PLOS Computational Biology* papers in genomics since 2017, we note that these are primarily papers describing improvements to existing software tools rather than introducing wholly new analysis techniques or algorithms. For example, BEAST 2.5 (Bouckaert and colleagues [2], cited 3,959 times according to Google Scholar as of August 2025) expanded upon BEAST, a software framework for Bayesian phylogenetic inference using MCMC, to now enable joint inference over multiple data types, non-tree models, and complex phylodynamics. Likewise, MUMmer4 (Marcias and colleagues [3], cited 2,080 times according to Google Scholar as of August 2025) improved upon MUMmer, a genome sequence aligner, to now accommodate genome sizes of any biologically realistic length, improve speed through parallel processing of input query sequences, and accommodate additional scripting languages. Such high citation counts reflect the impact of these works as well as the importance of their continual maintenance and ongoing development to meet the demands of increasingly large and complex datasets. Importantly, I believe by enabling such “derivative” work to be published in a peer-reviewed journal, *PLOS Computational Biology* serves as an integral mechanism to provide credit and career advancement opportunities to incentivize such software maintenance and sustain computational biology infrastructure.

## Future outlook

The first 10-year anniversary commentary 10 years ago provided a broad reflection on the journal’s first decade from the journal’s founders as well as speculations into the future [4]. Even 10 years ago, the journal’s founders wondered what would become of a computational biology journal once all biologists become computational biologists given the increasing size and complexity of biological datasets demanding computational analysis. In the next 10 years, I anticipate this demand for computational analysis of biological data will continue, resulting in the continued growth of the field. It will be interesting to see how this growth, perhaps into more diverse sectors and data modalities such imaging and temporal data, will influence the popularity of different programming languages, data sharing standards, as well as code and data-sharing infrastructure or even lead to the creation of new ones. I hope that journals like *PLOS Computational Biology* will continue to lead in the promotion of data and code accessibility for published works to move beyond data and code availability towards data and code reusability. However, it will be important for the field to broadly consider what incentive structures reinforced through policies and guidelines may encourage such practices.

I speculate this future will be heavily shaped by the wide-spread adoption of large language models (LLMs) and other deep learning AI tools such as coding agents. Perhaps LLMs and coding agents, much like graphical-user-interfaces, will help make standardized computational analyses more accessible to biologists, enabling improved degrees of tailoring even without programming experience but still demanding high level understanding for appropriate application. Perhaps in turn, computational biologists with programming experience will be better able to focus on developing new, bespoke analyses for unique data modalities or data representations not yet well represented in the training data. Whether other deep learning AI tools are developed and deployed in the editorial and publication process such as to help identify peer reviewers, spot image manipulation, or in the scientific and research process itself, I believe it will be up to the field to collectively and collaboratively develop cultural norms and guidelines that help ensure such tools augment our abilities, without replacing our intellectual capacity to define what is a scientific problem worth pursuing, how best to pursue that scientific problem, and how best to clearly communicate the resulting scientific advances in a manner that creates a robust foundation upon which we can build for future posterity. Ultimately, it will continue to be up to all of us as computational biologists to reflect on our best practices and values to help ensure that computational biology tools, be they deep learning AI or not, are develop and deployed in a manner that reflects and enacts these best practices and values.
